# Statistical Fragility of Surgical Clinical Trials in Orthopaedic Trauma

**DOI:** 10.5435/JAAOSGlobal-D-20-00197

**Published:** 2021-11-19

**Authors:** Lynn Ann Forrester, Kyle L. McCormick, Lisa Bonsignore-Opp, Liana J. Tedesco, Eric S. Baranek, Eugene S. Jang, Wakenda K. Tyler

**Affiliations:** From the Department of Orthopedic Surgery (Dr. Forrester, Dr. Tedesco, Dr. Baranek, Dr. Jang, and Dr. Tyler), Columbia University Irving Medical Center and the Columbia University Vagelos College of Physicians and Surgeons (Dr. McCormick and Bonsignore-Opp), New York, NY.

## Abstract

The Fragility Index (FI) and the Fragility Quotient (FQ) are powerful statistical tools that can aid clinicians in assessing clinical trial results. The purpose of this study was to use the FI and FQ to evaluate the statistical robustness of widely cited surgical clinical trials in orthopaedic trauma.

**Methods:**

We performed a PubMed search for orthopaedic trauma clinical trials in high-impact orthopaedics-focused journals and calculated the FI and FQ for all identified dichotomous, categorical outcomes.

**Results:**

We identified 128 studies with 545 outcomes. The median FI was 5, and the median FQ was 0.0482. For statistically significant and not statistically significant outcomes, the median FIs were 3 and 5, and the mean FQs were 0.0323 and 0.0526, respectively. The FI was greater than the number of patients lost to follow-up in most outcomes.

**Conclusions:**

The orthopaedic trauma literature is of equal or higher quality than research in other orthopaedic subspecialties, suggesting that other orthopaedic subspecialties may benefit from modeling their clinical trials after those in orthopaedic trauma.

In 2017, the American Academy of Orthopaedic Surgeons announced the launch of several centralized orthopaedic data registries, demonstrating a commitment to the production of high-quality research in orthopaedic surgery. This announcement was a reflection of ongoing discussions in the field concerning how to improve research quality in orthopaedic surgery. Recently, in orthopaedic trauma, substantial efforts have been made to coordinate research across multiple trauma centers. For example, the Major Extremity Trauma Research Consortium was established in September 2009 with funding from the Department of Defense in an effort to define best practice guidelines for orthopaedic trauma.^1^ Thus far, discussions regarding research quality in orthopaedic trauma have focused on concerns such as recycling of data, journal impact factor, level of evidence, and regional research bias.^2^ However, little attention has been devoted to evaluating the quality of outcomes reported in orthopaedic trauma clinic trials.

The *P* value is a powerful statistical tool that is commonly used to evaluate outcomes in research. However, the *P* value exclusively provides information relevant to the compatibility of data with a null hypothesis, while providing no information concerning effect size, strength of association, or applicability of a research outcome to a specific population.^3^ Walsh et al and other research groups have advocated for the use of alternative measures of statistical association such as the Fragility Index (FI) to act as a partner to the *P* value.^3-13^

The FI is a powerful tool that can aid clinicians in the assessment of clinical trial results. This metric is defined as the number of patients who would need to have an alternative outcome to convert a clinical trial result from statistically significant to not statistically significant. It is calculated by stepwise altering the outcome status of patients included in one study arm, with the goal of determining how many event changes would be necessary to switch the outcome from statistically significant (*P* < 0.05) to not statistically significant (*P* > 0.05) or vice versa. A smaller FI suggests less confidence in the strength of the outcome, as few events would need to change to alter the original observed result. Conversely, a larger FI inspires a higher amount of confidence in a result, as it would require many events to change its result. An additional metric used in conjunction with the FI is the Fragility Quotient (FQ), defined as the FI divided by study sample size. This statistical tool allows for interpretation of the FI in the context of a study's sample size, and typically, a lower FQ is associated with a more statistically robust study.

The FI for orthopaedic subspecialties is generally low, with reported fragility indices ranging from two to five.^4,6,7,12,14^ Thus far, one study by Parisien et al^16^ has evaluated the FI and FQ of the orthopaedic trauma literature. Although their study comprehensively analyzed studies published in the *Journal of Orthopaedic Trauma* (JOT) and in the *Journal of Bone and Joint Surgery* (JBJS), it did not evaluate the entirety of the orthopaedic trauma literature. In addition, their study evaluated all comparative studies, but did not focus on clinical trials, the studies that are primarily used in the establishment of clinical practice guidelines. Thus, the primary objective of this study was to use the FI and FQ to evaluate the statistical strength of widely cited surgical clinical trials in the orthopaedic trauma literature. Our hypothesis was that given the substantial efforts that have been made to improve the rigor and quality of clinical trials in orthopaedic trauma, the orthopaedic trauma literature likely has a higher FI and FQ, and thus more robust, reliable findings, when compared with other orthopaedic subspecialties. A secondary goal of this study was to identify what features of clinical trials are associated with greater statistical strength. We intend to use these findings to further inform discussions of how to improve the quality of the orthopaedic literature.

## Methods

### Study Design and Eligibility Criteria

We performed a systematic survey of clinical trials in orthopaedic trauma published in high-impact journals. To identify these studies, we first identified the highest-impact journals relevant to orthopaedic surgery. Using InCites *Journal Citation Reports*, we performed a search in a manner similar to previous work evaluating statistical fragility in healthcare research.^17-19^ We identified the top 25 highest-impact orthopaedic surgery journals. Next, we performed a search in PubMed for clinical trials published in the aforementioned journals. Our search included articles published from January 1, 2009, through March 15, 2019. We also applied the medical term trauma to identify trauma studies in the orthopaedics literature.

### Identification of Studies

After performing the search described above, we screened all titles for relevance to orthopaedic trauma. Then, we screened the abstracts of all remaining studies for surgical interventions and bone trauma; studies exclusively examining nonsurgical interventions were excluded from this analysis. We also simultaneously screened abstracts to ensure that studies evaluated human subjects and were published in English. Finally, as previously described in Walsh et al, we read each of the remaining studies and identified all dichotomous, categorical study outcomes that could be appropriately described using 2 × 2 contingency tables.^4^

### Study Characteristics

We collected the following information from each study that met the inclusion criteria: title, publication year, patient sample size, number of patients lost to follow-up, study outcomes, reported *P* value, and journal title. We then filled out a 2 × 2 contingency table for each dichotomous, categorical study outcome. Next, we used the InCites *Journal Citation Reports* to identify journal impact factor and number of journal citations and the National Institutes of Health iCite database to identify the relative citation ratio (RCR) for each study included in this analysis.^18,20^ Finally, we used the Web of Science to collect data on the number of citations for each of the articles evaluated in our study.^12^

### Calculation of Fragility Index and Fragility Quotient

Using the method previously described by Walsh et al,^13^ we calculated the FI for all categorical, dichotomous outcomes reported in the articles included in this study. We recalculated the *P* value for each outcome using the Fisher exact test. Using the group with fewer events, we switched events from one outcome to another, stepwise, until the calculated *P* value was greater than 0.05. Alternatively, if the recalculated *P* value was not statistically significant, we switched events in the smaller intervention group from one outcome to another, stepwise, until the calculated *P* value was less than 0.05. In each case, the smallest change in number of outcomes that was sufficient to obtain a *P* value that changed the outcome from significant to nonsignificant or vice versa was calculated as the FI for that outcome. Then, we calculated the corresponding FQ for all outcomes evaluated in this study by dividing the FI for each but the study sample size.

### Statistical Analysis

We analyzed descriptive statistics for each study and outcome included in our study. In addition, we used the Pearson correlation coefficient to determine associations between study variables and the Student *t*-test to analyze subpopulations within the study data. Analyses were performed using Microsoft Excel (2007) and SPSS (Version 19.0). To account for the fact that there were different numbers of outcomes in each study, we used the highest calculated FI from each article in all calculations comparing publication-level variables. This allowed us to avoid inappropriate weighting of studies with a higher number of outcomes in correlation calculations. We evaluated the following publication-level variables: patient sample size, RCR, publication year, number of article citations, journal impact factor, and number of journal citations.

## Results

### Study Selection

We identified 1314 articles in our initial search. After screening these titles and excluding studies according to the criteria described above, we selected 193 articles for further evaluation. After reading each article, we identified 128 articles with 545 dichotomous, categorical outcomes to be analyzed. Most these studies were prospective (96.1%), and most studies used randomization to allocate patients into intervention groups (88.2%).

### Characteristics of Trials and Outcomes

**Figure 1:**
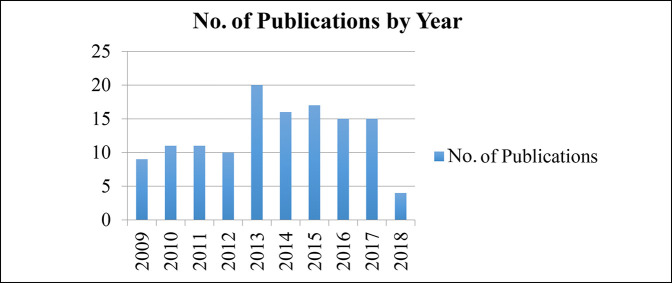
Bar graph showing the number of orthopaedic clinical trials published in each year of the study period.

The 128 identified studies were published between 2009 and 2019. Sixty-one of the studies were published from 2009 to 2013, and 67 of the studies were published between 2014 and 2018 (Figure 1). Studies were published in the following orthopaedics-focused journals: *Journal of Orthopaedic Trauma, Journal of Bone and Joint Surgery (American), Bone and Joint Journal, Spine, Clinical Orthopaedics and Related Research, Journal of Shoulder and Elbow Surgery, Journal of Hand Surgery (European), European Spine Journal, Foot and Ankle International, Acta Orthopaedica, Archives of Osteoporosis, Journal of the American Academy of Orthopaedic Surgeons, and Spine Journal* (Table 1).

**Table 1:** Number of Orthopaedic Trauma Clinical Trials Published in Orthopaedic Surgery Journals

Journals with Orthopaedic Trauma Clinical Trials	No. of Publications	Impact Factor (JCR 2018)
*Journal of Orthopaedic Trauma*	40	1.826
*Journal Bone and Joint Surgery* (American)	28	4.716
*Bone and Joint Journal*	13	4.301
*Spine*	10	2.903
*Clinical Orthopaedics and Related Research*	8	4.154
*Journal of Shoulder and Elbow Surgery*	8	2.865
*Journal of Hand Surgery (European)*	6	2.225
*European Spine Journal*	5	2.513
*Foot and Ankle International*	4	2.341
*Acta Orthopaedica*	3	3.217
*Archives of Osteoporosis*	1	2.469
*Journal of the American Academy of Orthopaedic Surgeons*	1	2.348
*Spine Journal*	1	3.196
Total	128	

JCR = *Journal Citation Reports*

Of the 545 dichotomous, categorical outcomes identified in these articles, 111 (20.4%) of these outcomes were primary, and 434 (79.6%) were secondary. Most outcomes were related to postoperative complications (69.5%), but outcomes related to patient pain/function (13.2%), radiographic findings (8.6%), mortality (4.6%), and patient satisfaction (2.6%) were reported as well.

The 128 trials examined in this study had a median sample size of 108.9 patients, (mean 73, range 17 to 964), and the median number of patients lost to follow-up per outcome was 3.0 (mean 16.2, range 0 to 551). The number of patients lost to follow-up was reported in 123 studies, and 525 outcomes were included in these articles. Of the trials examined, 51 reported that zero patients were lost to follow-up.

### Fragility Index and Fragility Quotient

The median FI for all outcomes was five (mean 5.7, range 1 to 32). A minority of these outcomes was statistically significant with a *P* value less than 0.05 (20.2%). For statistically significant outcomes, the median FI was three (mean 5, range 1 to 32), and the median FQ was 0.0323 (mean 0.0521, range 0.00391 to 0.361). Most outcomes were not statistically significant (79.8%); the median FI for these outcomes was five (mean 5.9, range 1 to 22), and the median FQ was 0.0526 (mean 0.0683, range 0.00146 to 0.296). The FI and FQ for statistically significant outcomes were significantly lower than the FI and FQ for outcomes that were not statistically significant (*P* = 0.05). In addition, the FI was greater than the number of patients lost to follow-up in 275 outcomes (53.3%), demonstrating that most orthopaedic trauma study outcomes would not have changed with more rigorous patient follow-up. As to be expected, a significant negative correlation was found between FI and *P* values for statistically significant outcomes (r = −0.511, *P* < 0.0001), and a significant positive correlation was found between FI and *P* values for outcomes that were not statistically significant (r = 0.3047, *P* < 0.0001). Similarly, a significant negative correlation was found between the FQ and *P* values for statistically significant outcomes (r = −0.459, *P* < 0.0001), and a significant positive correlation was found between the FQ and *P* values for outcomes that were not statistically significant (r = 0.260, *P* value < 0.0001).

Evaluation of publication-level variables showed that FI was positively correlated with patient sample size (r = 0.410, *P* < 0.00001), publication year (r = 0.177, *P* = 0.046), journal impact factor (r = 0.214, *P* = 0.015), and number of journal citations (r = 0.187, *P* = 0.034). No correlation was found between the FI and number of patients lost to follow up, RCR, or number of article citations. A significant positive correlation was found between the number of article citations and journal impact factor (r = 0.221, *P* = 0.012) and between patient sample size and journal impact factor (r = 0.136, *P* = 0.0125). However, no significant correlation was found between patient sample size and number of article citations.

## Discussion

This is the first study to examine the FI and FQ for surgical clinical trials in orthopaedic trauma. We found that the median FQ was 0.0323 for statistically significant outcomes and 0.0526 for outcomes that were not statistically significant. These findings suggest that if 3.2 or 5.3 patients of a sample size of 100, respectively, were to experience an alternative outcome, the study would have had a different overall result. We also found that the median FI was 3 for statistically significant outcomes and 5 for outcomes that were not statistically significant. These FIs are comparable to or higher than what has been previously observed in other orthopaedic subspecialties.^4,6,7,12,14^ In addition, we found the FI to be greater than the number of patients lost to follow-up in most outcomes, suggesting that most outcomes in orthopaedic trauma clinical trials would remain the same even with improved patient follow-up. Comparable studies evaluating statistical fragility of the orthopaedic literature have shown that the FI is frequently smaller than the number of patients lost to follow-up.^4,6,7,12,14^ These findings suggest that the orthopaedic trauma literature is at least equally or even more statistically robust than research in other orthopaedic subspecialties. Recent work has shown that studies informing the AAOS Clinical Practice Guidelines that are listed as having strong evidence have a median FI of 2. Thus, it is likely that other orthopaedic subspecialties may benefit from modeling their clinical trials after those in orthopaedic trauma.

A recent study by Parisien et al evaluated the FI and FQ of a series of publications in the orthopaedic trauma literature. This study evaluated 198 comparative articles published in JOT and JBJS between 1991 and 2013 and found that the mean FI and FQ for this subset of the orthopaedic trauma literature were 5 and 0.046, respectively. Although our findings are similar to those reported in the study by Parisien et al, our analysis differs in several key regards. First, we reviewed a later publication period (2009 to 2019) in an effort to produce an up-to-date estimate of the statistical fragility of the orthopaedic trauma literature. Second, we evaluated all publications in the top 25 highest-impact orthopaedic surgery journals. In our analysis, we identified 68 clinical trials published in JBJS and JOT and 60 clinical trials published in the other journals listed above. By limiting their search to JBJS and JOT, it is likely that the study by Parisien et al missed a substantial number of publications in other high-quality orthopaedic surgery journals. Given that the clinical trials published in the other journals listed above likely inform clinical practice of orthopaedic trauma surgeons, we felt that it was necessary to do a more comprehensive review of the literature to accurately assess the orthopaedic trauma literature as it currently stands. Finally, we limited our analysis to surgical clinical trials. Although there is utility in evaluating all comparative studies in the orthopaedic surgery literature, clinical trials are most often used in the creation of clinical practice guidelines. Thus, we felt that narrowing our analysis in this regard would accurately reflect the quality of the literature that informs current clinical practice.

Our secondary goal of this study was to identify study characteristics that are associated with increased statistical fragility. We found a positive correlation between FI and patient sample size (*P* < 0.00001), a finding consistent with the understanding that increasing patient sample size increases confidence in a study's findings. We also found positive correlations between FI and journal impact factor (*P* = 0.015) and number of journal citations (*P* = 0.034), providing evidence that higher-impact publications tend to publish data that are more statistically robust. In addition, we found a positive correlation between FI and publication year (*P* = 0.046), a finding consistent with our hypothesis that organized efforts to produce higher-quality orthopaedic trauma research have led to the production of more statistically robust literature.

The primary strength of this study was the methodology we used to search for clinical trials in orthopaedic trauma. This strategy allowed us to evaluate a large number of surgical clinical trials published in a number of high-quality orthopaedic surgery journals, facilitating rigorous characterization of the orthopaedic trauma literature. Limitations of this study include the fact that the FI can only be used to evaluate categorical, dichotomous outcomes. Thus, we were unable to evaluate the quality of outcomes comparing more than two interventions or outcomes that were continuous variables. The FI has utility in evaluating the research literature, but additional work needs to be done to identify statistical tools that can evaluate the quality of other elements of the orthopaedics literature.

## Conclusions

In this study, we found that the median FI for clinical trials in orthopaedic trauma was 5, and the median FIs for statistically significant and not statistically significant outcomes were 3 and 5, respectively. These FIs are comparable to or higher than what has been previously observed in other orthopaedic subspecialties. In addition, we found that most outcomes in orthopaedic trauma had an FI that was greater than the number of patients lost to follow-up. Research in other orthopaedic subspecialties had shown that the FI is more commonly smaller than the number of patients lost to follow-up. These findings suggest that the orthopaedic trauma literature has more statistical strength and is of higher quality than the literature produced by other orthopaedic subspecialties. Given that recent work has shown that studies informing the AAOS Clinical Practice Guidelines that are listed as having strong evidence have a median FI of 2, other orthopaedic subspecialties may benefit from modeling their clinical trials after those in orthopaedic trauma.
